# In Memoriam—Dr. Walter B. Farley

**Published:** 1899-07

**Authors:** 


### IN MEMORIAM—DR. WALTER B. FARLEY.

"Farley.—On May 25th, Dr. Walter B. Farley, aged 31 years. Funeral services Sunday afternoon at 2 o'clock, at the residence of his mother, Berwyn, Chester County, Pa."

The above notice appeared in the Philadelphia *Times* of May 27th, 1899. It announces the death of one of the brightest young physicians of Chester county, in the State of Pennsylvania.

Dr. Farley graduated from Hahnemann College, Philadelphia, in 1890. He immediately set up in practice at Berwyn, a village less than twenty miles from Philadelphia.

He was a strict homœopathist and a man of unimpeachable character.

If his earthly career had not been brought to a termination so soon, he would have become a prominent and successful physician and one of the leading doctors of Chester county.

He was a member of The International Hahnemannian Association and of The Medical Council of Philadelphia. The latter society was founded by the late Dr. Mahlon Preston with the object of affording professional advice and assistance to those of its members who may be treating difficult cases that baffle their skill.

Dr. Farley has a brother, Dr. Robert Farley, of Phœnixville, Chester county, Penna., well known in the profession as a successful practitioner of the strict methods of pure Homœopathy.

The deceased leaves a wife and one child to mourn his untimely removal from this earthly plane.

